# Coevolution of neoplastic epithelial cells and multilineage stroma *via* polyploid giant cells during immortalization and transformation of mullerian epithelial cells

**DOI:** 10.18632/genesandcancer.102

**Published:** 2016-03

**Authors:** Shiwu Zhang, Imelda Mercado-Uribe, Anil Sood, Robert C. Bast, Jinsong Liu

**Affiliations:** ^1^ Department of Pathology, Tianjin Union Medical Center, Tianjin, P.R. China; ^2^ Department of Pathology, University of Texas, MD Anderson Cancer Center, Houston, Texas, USA; ^3^ Department of Molecular and Cellular Oncology, University of Texas, MD Anderson Cancer Center, Houston, Texas, USA; ^4^ Department of Gynecologic Oncology, University of Texas, MD Anderson Cancer Center, Houston, Texas, USA; ^5^ Department of Cancer Biology, University of Texas, MD Anderson Cancer Center, Houston, Texas, USA; ^6^ Department of Center for RNA Interference and Non-Coding RNA, University of Texas, MD Anderson Cancer Center, Houston, Texas, USA; ^7^ Department of Experimental Therapeutics, University of Texas, MD Anderson Cancer Center, Houston, Texas, USA

**Keywords:** polyploid giant cells, multipotent cancer stem cells, stromal differentiation, RAS

## Abstract

Stromal cells are generally considered to be derived primarily from the host's normal mesenchymal stromal cells or bone marrow. However, the origins of stromal cells have been quite controversial. To determine the role of polyploidy in tumor development, we examined the fate of normal mullerian epithelial cells during the immortalization and transformation process by tracing the expression of SV40 large T antigen. Here we show that immortalized or *HRAS*-transformed mullerian epithelial cells contain a subpopulation of polyploid giant cells that grow as multicellular spheroids expressing hematopoietic markers in response to treatment with CoCl_2_. The immortalized or transformed epithelial cells can transdifferentiate into stromal cells when transplanted into nude mice. Immunofluorescent staining revealed expression of stem cell factors OCT4, Nanog, and SOX-2 in spheroid, whereas expression of embryonic stem cell marker SSEA1 was increased in *HRAS*-transformed cells compared with their immortalized isogenic counterparts. These results suggest that normal mullerian epithelial cells are intrinsically highly plastic, via the formation of polyploid giant cells and activation of embryonic stem-like program, which work together to promote the coevolution of neoplastic epithelial cells and multiple lineage stromal cells.

## INTRODUCTION

A carcinoma is a complex tissue composed of multiple cell types, including epithelial cancer cells as well as stromal cells that include fibroblasts, endothelial cells, and inflammatory cells [1-4]. Although it is well known that cancer epithelial cells and stromal cells interact extensively to form a critical network that sustains and regulates cancer growth, the development of cancer is generally attributed to clonal proliferation of transformed epithelial cells, resulting from accumulated genetic mutations of either somatic or adult stem cells. It is generally believed that mutational events lead to uncontrolled proliferation of epithelial cells and that stromal components of tumors are derived from the host's normal mesenchymal stroma or bone marrow-derived mesenchymal stem cells [1, 2]. Some studies, however, have demonstrated that endothelial cells can be derived from cancer cells [5-7], and other studies have shown that stromal components of tumors may not be derived entirely from the host's normal mesenchymal stroma or bone marrow [8, 9], suggesting that cancer cells are highly plastic and capable of generating stromal cells.

Polyploid giant cancer cells (PGCCs) are mononucleated or multinucleated cells with multiple genome copies that can be observed in cancer but that can also play an important role in many normal physiologic and nonmalignant pathologic processes [10-12]. Traditionally, PGCCs were considered to be senescent and not able to divide, since multiple mechanisms exist in animal cells to limit polyploid cell growth [13, 14]. However, data from several laboratories have shown that PGCCs are capable of generating daughter cells *via* budding [15-20]. We recently showed that PGCCs form *via* endoreplication in response to chemically induced stress; moreover, we found that these cells are capable of generating daughter cells *via* amitotic mechanisms such as budding, splitting, or bursting, similar to amitotic division used for asexual reproduction in yeast or protozoa [21]. In addition, we showed that PGCCs were tumorigenic in nude mice and capable of differentiation into multilineage stromal cells including erythroid cells expressing embryonic hemoglobin [21-23]. Thus, PGCCs may be novel multipotential stem cells that are capable of generating not only cancer cells (and thus may be a previously unrecognized key player in cancer development) but also stromal components.

In this study, we further examined the role of polyploid giant cells in immortalization and transformation, the earliest stages of tumor development before the tumors become life-threatening. We tracked the origin of mullerian epithelial cells by using a panel of well-defined genetically altered mullerian epithelial cell lines of fallopian tube or ovarian origin through serial introduction of SV40 T/t or the catalytic subunit of telomerase (hTERT) singly or in combination, followed by introduction of *HRAS* as described previously [24, 25].

## RESULTS

### Formation of polyploidy giant cells and spheroids in response to treatment with CoCl_2_

We examined the status of giant cells and spheroid formation by using a panel of fallopian tube or ovarian epithelial cells that were sequentially transfected with well-defined genetic elements, individually or in combination as described previously [21, 24, 25]. Primary cultured fallopian tubal epithelial cells (FTE187) were transfected with hTERT (FTE187hT), the catalytic subunit of human telomerase, alone or in combination with p53 knockdown (FTE187p53ihT), or in combination with SV40 (FTE187SV40hT); primary cultured fallopian tubal epithelial cells (FTE187) were also transfected with hTERT in combination with SV40 T/t and the *HRAS* oncogene (FTE187SV40hTHRAS) to make them progressively more tumorigenic. After treatment with CoCl_2_, giant cells were observed in all five of these cell lines but rarely in the untreated control fallopian tube epithelial cell 187. The total number of spheroids in each flask was measured in 10 fields, and averaged numbers were compared (Table 1). The number of spheroids increased with the number of genetic modifications introduced, which was positively correlated with the number of giant cells. The largest number of spheroids was observed in FTE187SV40hTHRAS cells (Table 1).

**Table 1:** Number of spheroids of series cell lines after CoCl_2_ treatment

Name of cell line	Average number of spheroids per field
FTE187 parental cells	1.30±0.21
FTE187hTERT	2.51±0.43
FTE187p53ihTERT	2.81±0.38
FTE187SV40hTERT	3.34±0.23
FTE187SV40hTHras	3.58±0.11

**Figure 1:**
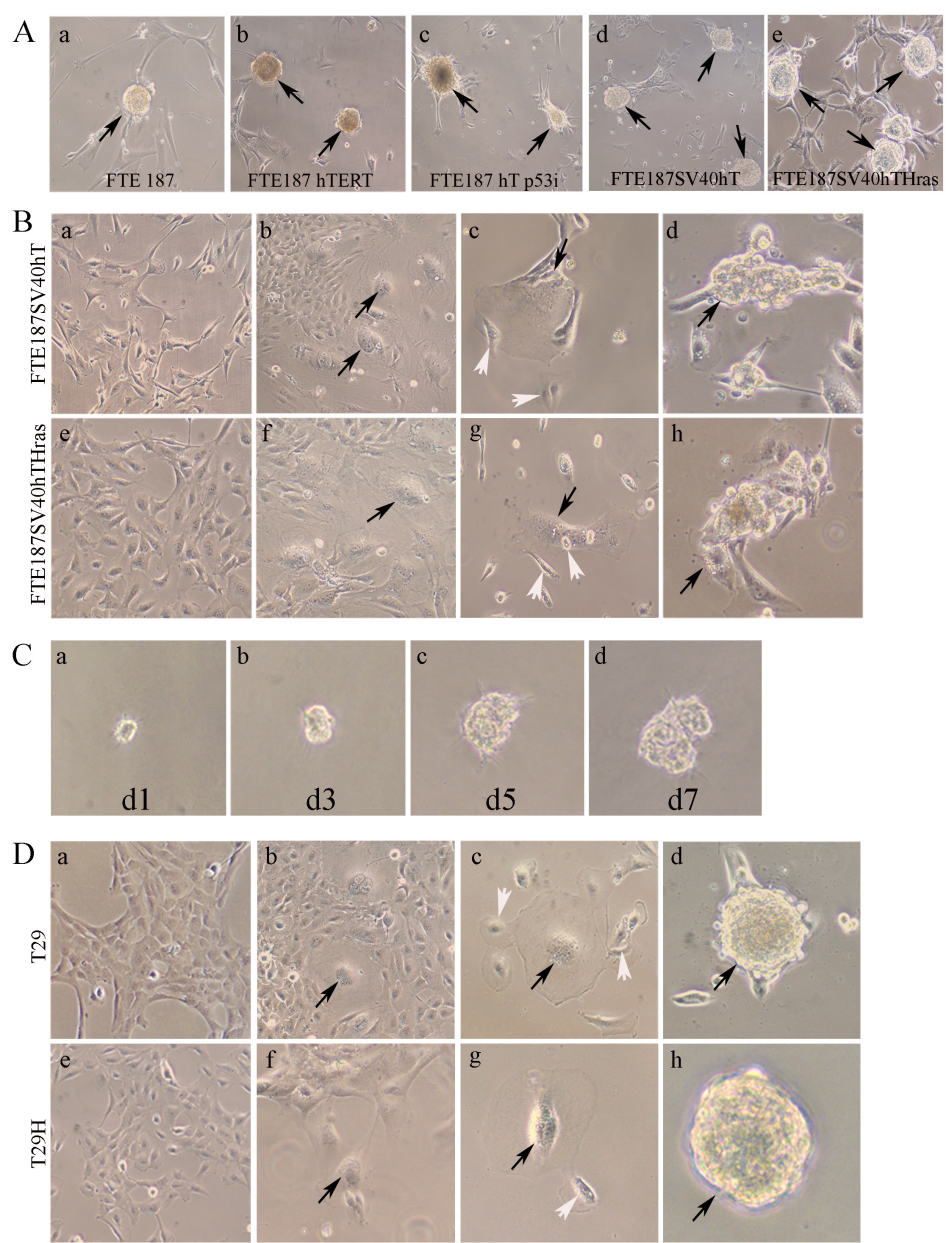
**A.** Formation of spheroids (black arrows) from stepwise genetically defined human fallopian tube epithelial cell lines after treatment with CoCl_2_ (×10). (a) Parental FTE187 cells; (b) FTE187hT immortalized with hTERT; (c) FTE187p53ihT cells; (d) FTE187SV40hT cells; and (e) FTE187SV40hTHras cells. **B.** Morphology of giant cells and spheroids derived from immortalized FTE187SV40hT (a-d) and *HRAS*-transformed isogenic line FTE187SV40hTHRAS cells (e-h) after treatment with CoCl_2_ (×10). (a,c) Control cells without CoCl_2_ treatment; (b,f) formation of giant cells after treatment with CoCl_2_ (black arrows); (c,g) daughter cells (white arrows) budded from giant cells (black arrows) after treatment of CoCl_2_; and (d,h) formation of spheroid after CoCl_2_ treatment (black arrows). **C.** Spheroid formed by a single FTE187SV40hTHras PGCC growing in Matrigel 7 days after seeding. d1, day 1; d3, day 3; d5, day 5; d7, day 7 (×20). **D.** Formation of giant cells and spheroid in immortalized ovarian epithelial cell T29 and HRAS-transformed isogenic line T29H (×10). (a,e) Control cells without CoCl_2_ treatment; (b,f) formation of giant cells (black arrows) after CoCl_2_ treatment; (c) daughter cells (white arrows) budded PGCCs (black arrows) after CoCl_2_ treatment; and (d,h) representative spheroids from CoCl_2_ treated cells.

Compared with the control (Figure 1Ba and e), treatment of FTE187SV40hT or FTE187SV40hTHRAS with CoCl_2_ led to an increase in the number of giant cells that can be recognized morphologically under light microscopy (Figure 1Bb and f). When these large cells were cultured with complete medium for 5-7 days, they generated small daughter cells *via* budding (Figure 1Bc and g). Also, individual giant cell grew into spheroids (Figure 1Bd and h). The spheroid morphology on matrigel derived from a representative single polyploidy giant cell generated from FTE187SV40hTHRAS after treatment with CoCl_2_ from different days is shown in Figure 1C.

Cells from the previously described immortalized ovarian epithelial cell line T29, generated by using SV40T/t and hTERT, and T29H, generated by the addition of *HRAS* to T29, were also used [24]. Similar results were found in immortalized human ovarian epithelial cell line T29 together with *HRAS*-transformed isogenic counterparts (T29H) after CoCl_2_ treatment. Control T29 (Figure 1Da) and T29H (Figure 1De) showed an epithelial morphology when they were cultured in regular media; also, increased numbers of giant cells survived after CoCl_2_ treatment (Figure 1Db and f), which can generate daughter cells *via* budding 7-10 days after treatment (Figure 1Dc and g). A single giant cell can grow into a spheroid when cultured in complete medium (Figure 1Dd and h).

### Acquisition of embryonic-like properties from single polyploid giant cell-derived spheroid *in vitro*

**Figure 2:**
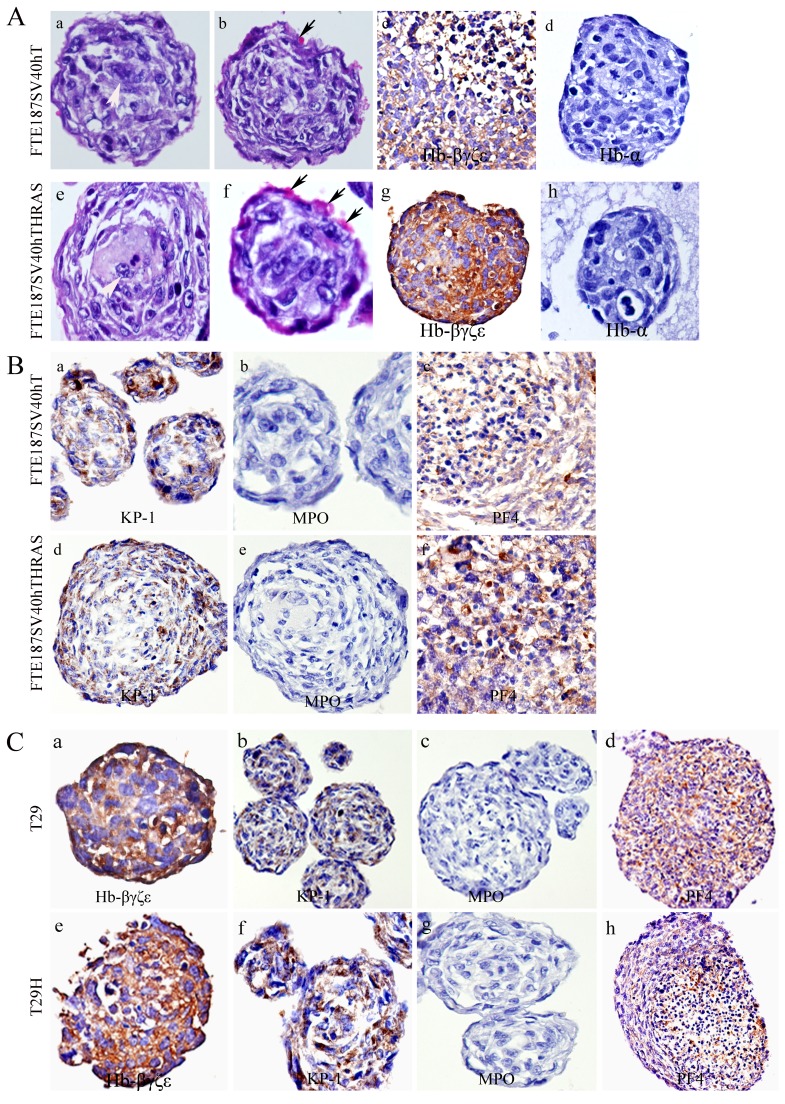
**A.** Morphology of and expression of hematopoietic differentiation in immortalized (FTE187SV40hT) and HRAS-transformed (FTE187SV40hTHRAS) cells in spheroids after treatment with CoCl_2_ (×10). (a and e) Morphology of spheroid (H&E) section with a giant cell in the center of spheroid (white arrows); (b and f) erythrocytes generated at the periphery of spheroids (black arrows); (c and g) hemoglobin-β/γ/ε/δ IHC expression; (d and h) negative expression of alpha hemoglobin. **B.** Expression of markers of hematopoietic lineage differentiation by FTE187SV40hT and FTE187SV40hTHras spheroids treated with CoCl_2_ (×10). (a, d) KP-1; (b, e) myeloperoxidase (MPO); (c,f) PF4; **C.** Multiple lineages of hematopoietic differentiation in spheroids in T29 and T29H after CoCl_2_ treatment (×10). (a,c) Hemoglobin-β/γ/ε/δ; (b,f) KP-1 stain; (c,g) MPO; and (d,h) PF4.

To determine the nature of cell types within the spheroids derived from FTE187SV40hT, FTE187SV40hTHRAS, T29, and T29H cells, the spheroids were formalin-fixed and embedded in paraffin blocks, which were then sectioned, made into slides, and subjected to pathologic and immunohistologic analysis. As shown in Figure 2Aa and e, the cells within the spheroid showed an undifferentiated morphology with a scattered giant cell in the center of the spheroid (white arrow). Of interest, erythroid cells (black arrow) were observed on the surface of the spheroids (Figure 2Ab and f). Cells within the spheroids showed strong positive staining for hemoglobin-β/γ/ε/δ (Figure 2Ac and g) and were negative for alpha hemoglobin (Figure 2Ad and h), demonstrating that spheroids from CoCl_2_ treatment have the potential for embryonic hematopoietic differentiation *in vitro*.

To further characterize the cell types within these spheroids, we examined the expression of a panel of markers that detect different lineages of hematopoietic differentiation, including KP-1 (macrophage), myeloperoxidase (MPO; neutrophil), and platelet factor 4 (PF4; platelet). As shown in Figure 2B, these spheroids were positive for KP-1 (Figure 2Ba and d) and PF4 (Figure 2Bc and f) staining and negative for MPO staining (Figure 2Bb and e), demonstrating that these spheroids have the potential to differentiate toward macrophages and platelet in immortalized or transformed fallopian tubal epithelial cells. Spheroids from immortalized ovarian epithelial cell T29 and *HRAS*-transformed ovarian epithelial cell T29H after CoCl_2_ treatment were also stained with the same markers as above and showed similar results (Figure 2C), demonstrating that the ability of *in vitro* differentiation from immortalized or *HRAS*-transformed ovarian epithelial cells was similar to that of immortalized ovarian epithelial cells.

### Multilineage differentiation in PGCC-derived xenograft

To determine the potential for multilineage differentiation from *RAS*-transformed FTE cells *in vivo*, 1×10^6^ FTE187SV40hTHRAS-derived PGCCs after CoCl_2_ treatment were injected into nude mice, and tumor tissue was collected for IHC staining. Since human fallopian tubal epithelial cells were immortalized with SV40 T antigen, thus T antigen provides a marker with which to trace the origin of these cells after differentiation *in vivo*. We examined the histopathologic features of the cell types that were positive for SV40 T antigen immunohistochemical staining (IHC) staining within the xenograft.

### Fibroblasts and adipose differentiation

**Figure 3:**
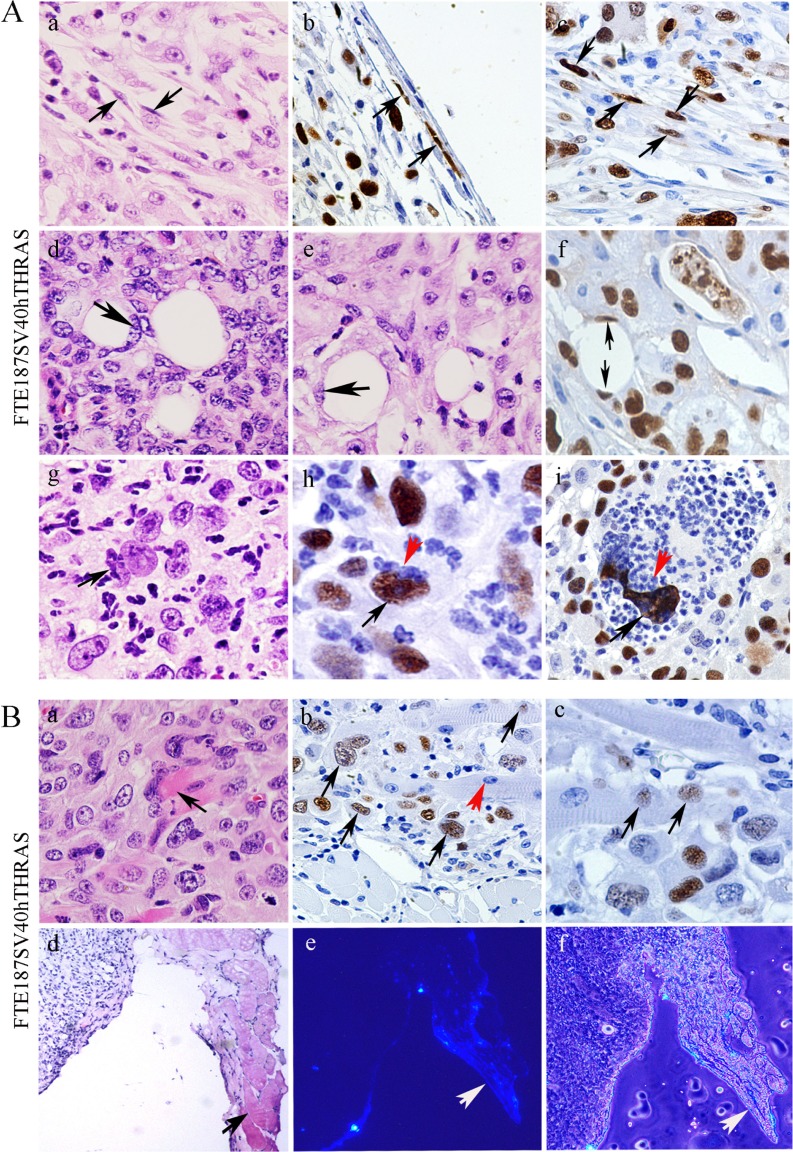
**A.** SV40 T antigen as an *in vivo* lineage tracing markers from differentiation of multilineage of stroma in tumor derived from FTE187SV40hTHras PGCCs in nude mice. (a) H&E staining shows fibroblasts (×20) (black arrows); (b, c) spindle-shaped fibroblast-like cells in the capsule of the tumor tissue positive for SV40 IHC staining (×20) (black arrows); (d and e) hematoxylin and eosin-stained adipocytes (×20) (black arrows); (f) SV40 T antigen IHC staining adipocytes (×20) (black arrows); (g) H&E staining shows neutrophil-like cells budding from FTE187SV40hTHRAS giant cells (×20) (black arrow); and (h and i) giant cells were positive for SV40 T antigen stain (black arrows) together with numerous SV40-negative budded neutrophil-like daughter cells (red arrows) (×20). **B.** Skeletal muscle differentiation. (a) H&E staining showed the skeletal muscle cells (×20) (black arrow); (b) positive SV40 stained nucleus in skeletal muscle (black arrows) mixed with negative SV40 stained nucleus (red arrow) (×20); (c) nuclei positive for SV40 T antigen staining in skeletal muscle (black arrow) (×20); (d) H&E staining of fresh tissue in tumor derived from injection of FTE187SV40hTHRAS PGCCs stained with H342 into nude mice (×10); (e) blue nuclei of skeletal muscle cells (white arrow) after H342 staining of the same fields shown in (d) (×10); and (f) merger of figures of (d) and (e) with phase contrast figures (×10).

There were scattered spindle-shaped cells distributed in the capsule or inside the tumor mass (Figure 3Aa). These spindle-shaped fibroblast-like cells underneath the tumor capsule (Figure 3Ab) and within the tumor (Figure 3Ac) were positive for SV40 T antigen-IHC staining. There were also a few adipocytes on histomorphologic examination (Figure 3Ad and e) as well as positive SV40 T antigen staining of their nuclei (Figure 3Af).

### Neutrophil-like differentiation

Neutrophil-like cells were also observed in giant cells and budded into multiple neutrophil-like cells (Figure 3Ag, black arrow). Of interest, although the giant nucleus stained positive for SV40 T antigen (black arrow), budded small daughter cells lost expression of T antigen (Figure 3Ah and I, red arrow). The underlying reason why expression of T antigen is lost in neutrophils is not clear.

### Skeletal muscle differentiation

Skeletal muscle differentiation was also observed in FTE187SV40hTHRAS xenografts (Figure 3Ba). The many nuclei of skeletal muscle cells were positive for SV40 T antigen staining (Figure 3Bb and c, black arrow), whereas some others lost such positivity (Figure 3Bb, red arrow). To further demonstrate the skeletal muscle differentiation of transformed fallopian tubal epithelial cells, we labeled the cells with Hoechst 33342 (H342) *in vitro* before injecting 1×10^6^ FTE187SV40hTHRAS-derived giant cells and daughter cells into nude mice. The mice were killed 6 weeks after injection, and the fresh tumor masses were removed and made into consecutive frozen sections for hematoxylin and eosin staining. Skeletal muscle was observed in hematoxylin and eosin-stained sections (Figure 3Bd); when the same section was examined under fluorescent microscopy, H342 stained blue nuclei were observed, further demonstrating that PGCCs derived from FTE187SV40hTHRAS were capable of muscle differentiation (Figure 3Be and f).

### Endothelial cell differentiation

**Figure 4:**
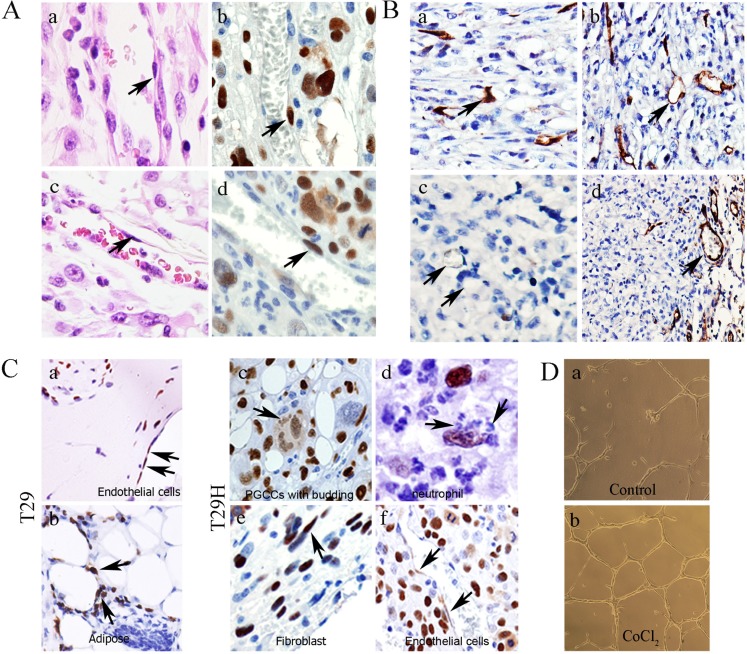
Endothelial differentiation in mullerian epithelial cell-derived xenografts **A.** Spindle-shaped endothelial cells lined blood vessels (H&E staining) (a and c) that are positive for SV40 T antigen stain (b and d) (black arrows) (×20). **B.** Human-specific CD34 and mouse-specific CD34 IHC staining of xenografts (×20). (a, b) Positive stain for human-specific CD34 (black arrows) and (c) negative for mouse-specific CD34 (black arrows). (d) Positive stain for mouse-specific CD34 vessels in surrounding mouse tissue (black arrows). **C.** Multilineage differentiation from spheroids from T29 and T29H injection into nude mice (×20). Endothelial cell (a) and adipose (b) differentiation in nodule from T29 injected site (black arrows). (c) Positive SV40 T antigen staining in giant cells and budding daughter cells, and in fibroblast (d), neutrophil-like cells (e), and endothelial cells (f) (black arrows). **D.** Tubal formation in T29H from CoCl_2_ treatment. (a) Control T29H; (b) T29H after CoCl_2_ treatment. All pictures were taken using a 10x objective lens; total magnification is 100×.

Hematoxylin and eosin staining showed that spindle-shaped endothelial cells lining the vessels (Figure 4Aa and c) were positive for SV40 T antigen staining (Figure 4Ab and d), demonstrating that PGCCs from *HRAS*-transformed fallopian epithelial cells were capable of differentiating toward endothelial cells. To further confirm endothelial differentiation, we stained the xenograft with use of a specific antibody against human but not mouse endothelium, comparing it with that of mouse-specific anti-CD34. Human-specific anti-CD34 staining highlighted both immature vessels from single cells (Figure 4Ba) and mature endothelium in vessels (Figure 4Bb), whereas similar vessels were largely negative for mouse-specific anti-CD34 staining (Figure 4Bc). These human-specific CD34-positive vessels were observed inside the tumor mass, while vessels immediately underneath the capsule from surrounding mouse tissue were positive for mouse-specific CD34 (Figure 4Bd). These results demonstrated that an *HRAS*-transformed xenograft was capable of differentiation toward endothelial cells.

The above results were further confirmed in immortalized ovarian epithelial cell lines T29 and *HRAS*-transformed isogenic T29H cells. T antigen-stained endothelial cells (Figure 4Ca) and adipose (Figure 4Cb) were observed within the xenograft from injected T29-derived giant cells; whereas SV40 T antigen-stained cells were found in multinucleated giant cells together with budding small daughter cells (Figure 4Cc, black arrow giant cells with budding neutrophil-like cells that lost T antigen staining (Figure 4Cd), ), spindle-shaped fibroblasts located in the capsule of tumor tissue (Figure 4Ce), as well as T antigen staining positive endothelial-like cells (Figure 4Cf). We also compared the capacity of T29H cells to form tubes, a characteristic of endothelial cells, before and after CoCl_2_ treatment *in vitro*. The control cells (Figure 4Da) produced a few fully formed tube-like structures, but the T29H cells treated with CoCl_2_ formed more intact tube-like structures than did the control cells (Figure 4Db), demonstrating that CoCl_2_ can induce endothelial cell differentiation from *HRAS*-transformed ovarian epithelial cells.

### Activation of expression of embryonic stem cell markers in spheroid

**Figure 5:**
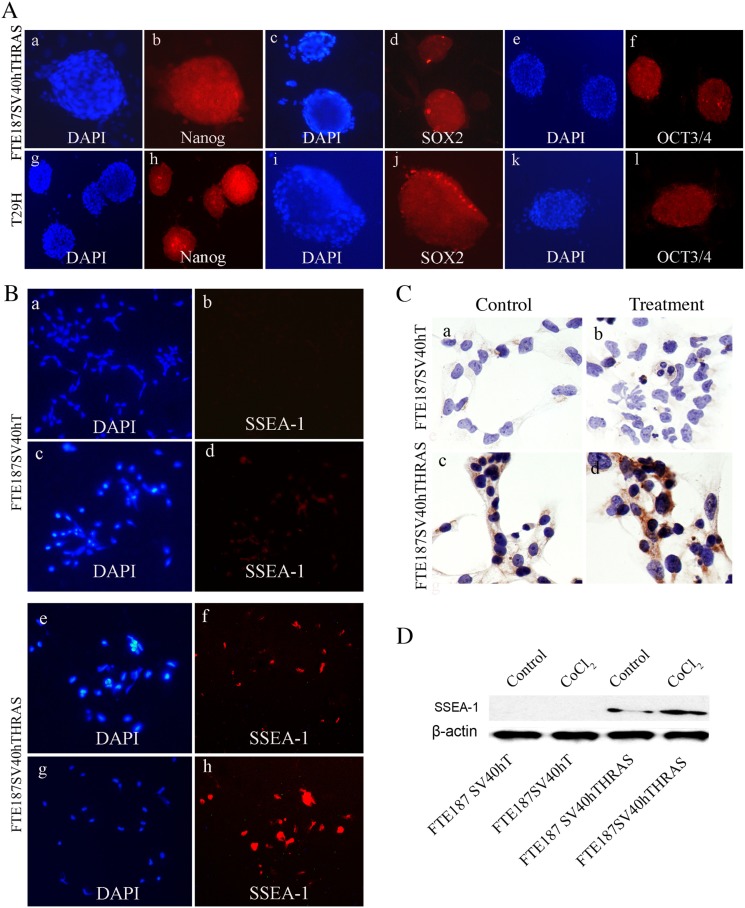
**A.** Expression of core stem cell factors OCT3/4, Nanog, and SOX2 in spheroid-derived FTE187SV40hTHRAS and T29H cells. The DNA was stained with DAPI (×10). **B.** Expression of SSEA-1 by immunofluorescence in FTE187SV40hT and FTE187SV40hTHRAS cells in the absence or presence of CoCl_2_ treatment. The DNA was stained with DAPI (×10). **C.** Expression of SSEA-1 by immunocytochemical staining in FTE187SV40hT and FTE187SV40hTHRAS cells in the absence or presence of CoCl_2_ (×20). **D.** Western blot of SSEA-1 expression in FTE187SV40hT and FTE187SV40hTHRAS cells in the absence or presence of CoCl_2_ treatment.

Since the ability to differentiate into multilineage of stromal cells is a property of embryonic-like stem cells, we further examined expression of the core embryonic stem cell markers Nanog, SOX2, and OCT3/4 from spheroidsof FTE187SV40hTHRAS and T29H giant cells after CoCl_2_ treatment. The nuclei of spheroids were all stained blue by DAPI, there were scattered cells positive for Nanog, SOX2, and OCT3/4 immunofluorescent staining in FTE187SV40hTHRAS (Figure 5Ab, d and f) and T29H spheroids (Figure 5Ah, j and l). Of interest, SSEA-1, a marker for early differentiation of human embryonic stem cells and a cancer stem cell marker in human and mouse brain tumors [26], was positive in *HRAS*-transformed cells but not in immortalized fallopian tube cells by immunofluorescent staining (Figure 5B), immunocytochemical staining (Figure 5C), and Western blotting (Figure 5D); no major difference in SSEA-1 expression was detected before or after CoCl_2_ treatment. These results indicated that HRAS, rather than CoCl_2_, is the key element in activating the expression of embryonic stem cell marker SSEA-1.

## DISCUSSION

Initiation of cancer requires coordinated interactions of multiple signals that disable normal epithelial cell homeostasis and lead to uncontrolled growth [27, 28]. To dissect such a complicated signaling network in cancer development, the process of immortalizing cells of human origin with SV40 T/t and hTERT and then transforming these cells with *HRAS* was first developed in 1999 to create a genetically well-defined model [29]; this model proved to be tremendously useful in understanding the mechanisms involved in the transformation of human cells [24, 28-30]. Here, we provide strong evidence that both immortalized and tumorigenic fallopian and ovarian epithelial cells are highly plastic and are capable of differentiation toward benign lineages of stromal cells. Such plasticity can be augmented by HRAS or CoCl_2_. These treated cells tend to grow as giant cells, are capable of forming spheroids *in vitro*, and differentiate into multilineage stromal cells in nude mouse xenografts. Our studies reported here provide further support of our recent findings that giant cells have embryonic-like properties [21-23].

Our results demonstrate that *HRAS*-transformed fallopian tube epithelial cells can differentiate into multilineage stromal cells to create an tumor-derived intrinsic microenvironment, including fibroblasts, adipocytes, neutrophils, erythroid cells, endothelial cells, and skeletal muscle during tumor development. This conclusion was also supported by results from several other laboratories. It has been shown that RAS is capable of activating stem-like properties [31, 32]. A recent study has demonstrated that tumor-initiating cells can be derived from *RAS*-induced multinucleated giant cells in melanoma [33]. An embryonic stem cell network was activated in giant cancer cells derived from mutant cancer lines [34]. Other investigators have reported that transformed fibroblasts are hierarchically organized and acquire stem cell properties [35]. Furthermore, SSEA-1 was found to act as a cancer stem cell marker to initiate and maintain tumor growth and is an enrichment marker for tumor-initiating cells in glioblastoma [26, 35]. These studies, along with ours, all support the conclusion that RAS activates embryonic-like stem cell programming *via* polyploidy giant cells during malignant transformation.

In summary, our data support the finding that giant cells play a critical role in tumor initiation. Formation of polyploidy facilitates coevolution of both neoplastic epithelial cells and stromal cells, leading to the formation of cancer.

## MATERIALS AND METHODS

### Tissue culture of immortalized and RAS-transformed mullerian epithelial cell lines

Cells from the primary human ovarian epithelial cell line T29 and its *HRAS*-transformed counterparts *HRAS* (T29H) were cultured by a method described previously [24]; cells from the fallopian tube epithelial cell line 187 (FTE187) were infected sequentially with retroviruses containing pBabe-hygro-hTERT (the catalytic subunit of human telomerase, FTE187hT) and pBabe-puro-p53 small-interfering RNA against *p53* mRNA (FTE187p53ihT). FTE187hT also was infected sequentially with retroviruses containing pBabe-zeo-SV40 early region (FTE187SV40hT) and pBabe-puro-HRASV12 (FTE187SV40hTHRAS) as described previously [25]. The resulting FTE187, FTE187hT, and FTE187p53ihT cell lines were maintained in Medium 199/MCDB 205 (1:1; Sigma-Aldrich Co.) supplemented with 10% FBS (Intergen), 10 ng/mL epidermal growth factor (Sigma-Aldrich), and 100 U/mL penicillin/streptomycin (Sigma-Aldrich), referred to as normal ovarian epithelial cell culture (NOE) medium. FTE187SV40hT and FTE187SV40hTHRAS were cultured with NOE cell culture medium without epidermal growth factor (referred to as regular medium).

### Induction of polyploid giant cells and spheroids with CoCl_2_

The five fallopian tube epithelial cell lines, parental FTE187, FTE187hT, FTE187p53ihT, FTE187SV40hT, and FTE187SV40hTHRAS, and four ovarian epithelial cell lines T29, T29H, T80, and T80H, were cultured with NOE medium or regular medium. When the confluency of all five cell lines reached 90%, 150 μM cobalt chloride (CoCl_2_, Sigma-Aldrich) was added to the medium in each flask for 24-48 hours (for detailed information used in the treatment of different cell lines, see Table S1). After the cells recovered from CoCl_2_ treatment and confluency again reached 90%, they were treated with CoCl_2_ once again. The cells were then rinsed with 1´ phosphate buffered saline (PBS) and cultured with complete medium. This treatment killed most of the normal-sized cells, whereas a few surviving giant cells could be identified with a light microscope (1×71; Olympus). After the cells recovered from the first CoCl_2_ treatment after 10-14 days, they were treated with CoCl_2_ for a second or third time. A portion of the PGCCs grew into spheroids in complete medium after trypsinization. Spheroids in 10 fields for the panel fallopian tube epithelial cell 187-derived cell lines with stepwise genetic modifications were counted, and the averaged numbers were used to compare the capacities of the cell lines to form spheroids.

### Spheroid formation by single FTE187SV40hTHras PGCCs *in vitro*

BD Matrigel matrix (BD Biosciences, San Jose, CA, USA) was thawed overnight at 4°C. Next, 1,000 FTE187SV40hTHras PGCCs derived *via* treatment with CoCl_2_ were thoroughly dispersed and cultured in a mixture of Matrigel and regular medium at a 1:1 ratio. These PGCCs were seeded at a total volume of 100 mL on a 96-well plate on ice. This cell mixture with Matrigel was solidified at 37°C for 10 min, and 0.1 mL of regular medium was added to the wells. The complete medium was replenished every 48 h. Single PGCCs in the matrigel were labeled and photographed every other day.

### Paraffin embedding of blocks of spheroids

The spheroids generated by the method described above were detached from the flask walls by vigorous pipetting. Samples of medium containing spheroids were put into 15-mL centrifuge tubes and centrifuged at 100 *g* for 5 minutes. The supernatant was removed, and the pellet was moved to a 1.5-mL vial. Ethanol (70%, 1 mL) was added to the pellet to fix the spheroids, and 50 mL of eosin was added to the vial to track the spheroids. The resulting spheroid samples were dehydrated in a graded ethanol series (70%, 80%, 95%, and 100% for 15 minutes per grade). The vials were then infiltrated serially with acetone, absolute xylene, a mixture of 50% xylene and 50% paraffin, and purified paraffin at 65°C for 15 minutes each. Finally, the spheroids were embedded in paraffin and sectioned for analysis.

### Immunofluorescence staining of spheroids

Spheroids formed by treatment of giant cells were detached from the walls of the flasks by pipetting. The medium containing the spheroids was subjected to centrifugation at 400 *g* for 5 minutes to pellet the spheroids. The spheroids were attached to coverslips after culture with complete medium for several hours and then were fixed in ice-cold acetone for 10 minutes. The samples were washed in Tween-20 (TBST) three times for 5 minutes per washing and were incubated with 1% bovine serum albumin (BSA) in PBST for 30 minutes to block unspecific binding of the antibodies. Without further washing, the samples were successively incubated with primary and secondary antibodies in PBST with 1% BSA in a humidified chamber for 1 hour at room temperature. The spheroids were stained with 4¢6-diamidino-2-phenylindole (DAPI) for 1 minute and observed with a fluorescence microscope (Eclipse TE 2000-U; Nikon).

### Hematoxylin and eosin, immunohistochemical (IHC), and immunocytochemical staining

For hematoxylin and eosin staining, 4-mm sections from formalin-fixed, paraffin-embedded spheroids were deparaffinized and rehydrated and then counterstained with hematoxylin for 1 minute and eosin for 2 minutes. The sections were then dehydrated and mounted with coverslips in preparation for microscopic examination. IHC and immunocytochemical staining were performed by using the avidin-biotin-peroxidase method, as described previously [36].

For IHC staining, the formalin-fixed and paraffin-embedded tissue was deparaffinized in xylene and rehydrated through a graded series of alcohols. The sections were washed with 1×PBS and then subjected to antigen retrieval in 0.01 M sodium citrate buffer (pH 6.0) in an autoclave for 10 minutes.

For immunocytochemical staining, the cells were grown on glass coverslips; when they reached 70% confluency, the slides were washed with PBS and fixed with pure cold acetone for 10 minutes on ice. The endogenous peroxidase activity was blocked with 0.3% hydrogen peroxide, nonspecific protein binding was blocked with 1.5% normal goat serum, and the sections were incubated with primary antibodies overnight at 4°C in a humidified chamber (for detailed antibody information, see Table S2). After cells were allowed to react with biotinylated goat anti-rabbit IgG for 20 minutes, the signal was detected with the labeled streptavidin-biotin system in the presence of chromogen 3,3′-diaminobenzidine or alkaline phosphatase. The nuclei were counterstained with hematoxylin, and the sections were dehydrated and mounted with coverslips.

### Western blot analysis

Western blot analyses were done as described previously [24]. Cell extracts were subjected to lysis in ice-cold buffer. The protein concentrations were determined, and the proteins were separated on a 10% sodium dodecyl sulfate polyacrylamide gel and transferred to a polyvinylidene fluoride membrane (Amersham Hybond-P PVDF Membrane; GE Healthcare). Afterward, the membranes were blocked with 5% nonfat milk in 1×tris-buffered saline solution with 0.1% TBST for 1 hour at room temperature and washed with 1×TBST three times. They were incubated with the appropriate primary antibody overnight at 4°C and then with the appropriate secondary antibody for 1 hour at room temperature on a rocking platform. Expression of various proteins, including stage-specific embryonic antigen-1 (SSEA-1) and H-ras, was measured by using mixed ECL Plus reagents (RPN2132OL/AK, GE Life Sciences Co.) and developed by using an X-OMAT 2000 film processor (Kodak). HSP90 was used as a protein-loading control. The antibodies used are described in Table S2.

### Tubal formation assay in cell culture

BD Matrigel matrix (356237; BD Biosciences) was thawed overnight at 4°C. Matrigel (50 mL per well) was added to a precooled 48-well culture plate, which was placed in a 37°C incubator for 30 minutes. The FTE187SV40hTHRAS cells and FTE187SV40hTHRAS cells that recovered from CoCl_2_ treatment were prepared in complete regular medium (5,000 cells per mL), and 500 μL of each cell suspension was added to a Matrigel-coated well with three repeat wells for each cell type. The plate was incubated in a humidified 5% CO_2_ atmosphere at 37°C. Ten hours later, the well contents were examined under a microscope for vessel-like structures.

### Nuclear staining of giant cells with Hoechst 33342

Giant cells derived from FTE187SV40hTHRAS were induced as already described, stained with Hoechst 33342 (H342; no. B2261; Sigma) at a final concentration of 2 μg/mL, and incubated at 37°C for 1 hour. The medium was changed to complete medium. The flask was wrapped with foil and kept in the incubator with normal culturing conditions. The medium was changed once a week. Two to three weeks later, the giant cells had recovered from H342 toxicity and had generated daughter cells, and the giant cells and daughter cells with a blue nucleus (1×10) were injected into nude mice. About 6 weeks later, the xenograft size had reached 0.8 cm. The mice were killed, and the tumor masses were removed and frozen-sectioned for observation.

### Tumor growth in nude mice

The giant cells (1×10) from FTE187SV40hTHRAS, T29, and T29H were trypsinized and then subjected to centrifugation at 400 *g* for 5 minutes. The supernatant was removed from each sample, and 1× PBS (0.1 mL) was added to resuspend the pellet. The giant cells, together with a mixture of PBS buffer (0.1 mL) and Matrigel (0.1 mL), were drawn into the syringes and kept on ice before injection. The giant cells were then injected subcutaneously into the flanks of 6-week-old nude mice, and 5 mice were used for each kind of cell line. The mice were kept in a pathogen-free environment and checked every 2 days for 2 months. After the 2-month monitoring period, each mouse that had been injected with FTE187SV40hTHRAS and T29H had developed a 1-cm tumor at the injection site, and there was a 0.4-cm-diameter nodule at the sites that had been injected with T29-derived giant cells. The mice were killed, and the tumors were removed and fixed in 10% formalin for routine histologic examination and IHC staining. The care and use of the mice was approved by the Institutional Animal Care and Use Committee at The University of Texas MD Anderson Cancer Center.

### Statistical analysis

Statistical analysis was performed with use of SPSS statistical analysis software (SPSS, Chicago, IL). A *P*-value of less than 0.05 was considered statistically significant. The statistical significance of the PGCC numbers was calculated by the Student *t* test. Data were reported as mean ± standard deviation.

## SUPPLEMENTARY MATERIAL TABLES



## References

[1] Junttila MR, de Sauvage FJ (2013). Influence of tumour microenvironment heterogeneity on therapeutic response. Nature.

[2] Plaks V, Kong N, Werb Z (2015). The cancer stem cell niche: how essential is the niche in regulating stemness of tumor cells?. Cell Stem Cell.

[3] Zhang J, Liu J (2013). Tumor stroma as targets for cancer therapy. Pharmacol Ther.

[4] Schauer IG, Sood AK, Mok S, Liu J (2011). Cancer-associated fibroblasts and their putative role in potentiating the initiation and development of epithelial ovarian cancer. Neoplasia.

[5] Ricci-Vitiani L, Pallini R, Biffoni M, Todaro M, Invernici G, Cenci T, Maira G, Parati EA, Stassi G, Larocca LM, De Maria R (2010). Tumour vascularization via endothelial differentiation of glioblastoma stem-like cells. Nature.

[6] Soda Y, Marumoto T, Friedmann-Morvinski D, Soda M, Liu F, Michiue H, Pastorino S, Yang M, Hoffman RM, Kesari S, Verma IM (2011). Transdifferentiation of glioblastoma cells into vascular endothelial cells. Proc Natl Acad Sci U S A.

[7] Wang R, Chadalavada K, Wilshire J, Kowalik U, Hovinga KE, Geber A, Fligelman B, Leversha M, Brennan C, Tabar V (2010). Glioblastoma stem-like cells give rise to tumour endothelium. Nature.

[8] Bussolati B, Grange C, Sapino A, Camussi G (2009). Endothelial cell differentiation of human breast tumour stem/progenitor cells. J Cell Mol Med.

[9] Jia L, Zhang S, Ye Y, Li X, Mercado-Uribe I, Bast RC, Liu J (2012). Paclitaxel inhibits ovarian tumor growth by inducing epithelial cancer cells to benign fibroblast-like cells. Cancer Lett.

[10] Comai L (2005). The advantages and disadvantages of being polyploid. Nat Rev Genet.

[11] Edgar BA, Zielke N, Gutierrez C (2014). Endocycles: a recurrent evolutionary innovation for post-mitotic cell growth. Nat Rev Mol Cell Biol.

[12] Fox DT, Duronio RJ (2013). Endoreplication and polyploidy: insights into development and disease. Development.

[13] Vakifahmetoglu H, Olsson M, Zhivotovsky B (2008). Death through a tragedy: mitotic catastrophe. Cell Death Differ.

[14] Ganem NJ, Pellman D (2007). Limiting the proliferation of polyploid cells. Cell.

[15] Erenpreisa J, Kalejs M, Ianzini F, Kosmacek EA, Mackey MA, Emzinsh D, Cragg MS, Ivanov A, Illidge TM (2005). Segregation of genomes in polyploid tumour cells following mitotic catastrophe. Cell Biol Int.

[16] Erenpreisa JA, Cragg MS, Fringes B, Sharakhov I, Illidge TM (2000). Release of mitotic descendants by giant cells from irradiated Burkitt's lymphoma cell line. Cell Biol Int.

[17] Walen KH (2006). Human diploid fibroblast cells in senescence; cycling through polyploidy to mitotic cells. In Vitro Cell Dev Biol Anim.

[18] Walen KH (2005). Budded karyoplasts from multinucleated fibroblast cells contain centrosomes and change their morphology to mitotic cells. Cell Biol Int.

[19] Walen KH (2004). Spontaneous cell transformation: karyoplasts derived from multinucleated cells produce new cell growth in senescent human epithelial cell cultures. In Vitro Cell Dev Biol Anim.

[20] Sundaram M, Guernsey DL, Rajaraman MM, Rajaraman R (2004). Neosis: a novel type of cell division in cancer. Cancer Biol Ther.

[21] Zhang S, Mercado-Uribe I, Xing Z, Sun B, Kuang J, Liu J (2014). Generation of cancer stem-like cells through the formation of polyploid giant cancer cells. Oncogene.

[22] Zhang S, Mercado-Uribe I, Liu J (2013). Generation of erythroid cells from fibroblasts and cancer cells in vitro and in vivo. Cancer Lett.

[23] Zhang S, Mercado-Uribe I, Liu J (2014). Tumor stroma and differentiated cancer cells can be originated directly from polyploid giant cancer cells induced by paclitaxel. Int J Cancer.

[24] Liu J, Yang G, Thompson-Lanza JA, Glassman A, Hayes K, Patterson A, Marquez RT, Auersperg N, Yu Y, Hahn WC, Mills GB, Bast RC (2004). A genetically defined model for human ovarian cancer. Cancer Res.

[25] Shan W, Mercado-Uribe I, Zhang J, Rosen D, Zhang S, Wei J, Liu J (2012). Mucinous adenocarcinoma developed from human fallopian tube epithelial cells through defined genetic modifications. Cell Cycle.

[26] Son MJ, Woolard K, Nam DH, Lee J, Fine HA (2009). SSEA-1 is an enrichment marker for tumor-initiating cells in human glioblastoma. Cell Stem Cell.

[27] Hanahan D, Weinberg RA (2011). Hallmarks of cancer: the next generation. Cell.

[28] Hahn WC, Weinberg RA (2002). Rules for making human tumor cells. N Engl J Med.

[29] Hahn WC, Counter CM, Lundberg AS, Beijersbergen RL, Brooks MW, Weinberg RA (1999). Creation of human tumour cells with defined genetic elements. Nature.

[30] Elenbaas B, Spirio L, Koerner F, Fleming MD, Zimonjic DB, Donaher JL, Popescu NC, Hahn WC, Weinberg RA (2001). Human breast cancer cells generated by oncogenic transformation of primary mammary epithelial cells. Genes Dev.

[31] Ischenko I, Zhi J, Moll UM, Nemajerova A, Petrenko O (2013). Direct reprogramming by oncogenic Ras and Myc. Proc Natl Acad Sci U S A.

[32] Moon BS, Jeong WJ, Park J, Kim TI, Min do S, Choi KY (2014). Role of oncogenic K-Ras in cancer stem cell activation by aberrant Wnt/beta-catenin signaling. J Natl Cancer Inst.

[33] Leikam C, Hufnagel AL, Otto C, Murphy DJ, Muhling B, Kneitz S, Nanda I, Schmid M, Wagner TU, Haferkamp S, Brocker EB, Schartl M, Meierjohann S (2015). In vitro evidence for senescent multinucleated melanocytes as a source for tumor-initiating cells. Cell Death Dis.

[34] Salmina K, Jankevics E, Huna A, Perminov D, Radovica I, Klymenko T, Ivanov A, Jascenko E, Scherthan H, Cragg M, Erenpreisa J (2010). Up-regulation of the embryonic selfrenewal network through reversible polyploidy in irradiated p53-mutant tumour cells. Exp Cell Res.

[35] Scaffidi P, Misteli T (2011). In vitro generation of human cells with cancer stem cell properties. Nat Cell Biol.

[36] Yang G, Chang B, Yang F, Guo X, Cai KQ, Xiao XS, Wang H, Sen S, Hung MC, Mills GB, Chang S, Multani AS, Mercado-Uribe I, Liu J Aurora kinase A promotes ovarian tumorigenesis through dysregulation of the cell cycle and suppression of BRCA2. Clin Cancer Res.

