# Supplementary material for: Coevolution of neoplastic epithelial cells and multilineage stroma via polyploid giant cells during immortalization and transformation of mullerian epithelial cells
Source: Genes Cancer. 2016 Mar;7(3-4):60–72. doi: 10.18632/genesandcancer.102 (PMC4918945; doi:10.18632/genesandcancer.102)
Supplement: Supplementary file 1 [file ganc-07-060-s001.pdf]

Coevolution of neoplastic epithelial cells and multilineage stroma *via* polyploid giant cells during immortalization and transformation of mullerian epithelial cells – Zhang et al

Supplementary Tables

Table S1: Concentration and duration of $CoCl_2$ treatment in various ovarian and fallopian tubal epithelial cell lines; all cell lines were treated with 150 μM $CoCl_2$.

Cell line	Duration of induction (hours)
FTE187	24
FTE187hTERT	24
FTE187p53ihTERT	24
FTE187SV40hT	48
FTE187SV40hTHRAS	48
T29	48
T29H	48
T80	48
T80H	48

Table S2**:** Antibody information used in our article

**Antibody name**	**Company (Cat number)**	**Dilution**	**Anti-rabbit or -mouse**
HSP-90	Epitomics	1:1,000 (WB)	Rabbit monoclonal antibody
Anti-hemoglobin aeta antibody	Fitzgerald 70R-1234	1: 250 (IHC)	R
Anti-fetal hemoglobin	Novus Biologicals NB110-41084	1:200 (IHC)	G
Anti-β/γ/δ/ε hemoglobin	Santa Cruz Biotechnology, INC (N-19) sc-22718	1:100 (IHC)	G
Anti-hemoglobin alpha chain	Epitomics (EPR3608)	1:100 (IHC)	Rabbit monoclonal antibody
β-catenin	Cell Signaling Technology (no. 2849)	1:10 (ICC)<br>1:10 (IF)	M
Anti-Nanog	Cell signaling	1:100 (IF)	M
Anti-SOX2	R&D system	1:100 (IF)	M
SSEA	DAKO	1:100(IF)<br>1:100(ICC)	M
SV40	Santa Cruz	1:100(IHC)	M
Human-specific CD34	Abcam	1:100(IHC)	R
Mouse-specific CD34	Abcam	1:100(IHC)	Rat monoclonal
PF4	Novus Biologicals	1:100(IHC)	M
KP1	Santa Cruz	1:100(IHC)	M
MPO	Epitomics	1:100(IHC)	Rabbit monoclonal antibody

WB, Western blot; M, mouse; R, rabbit; G, goat; IF, immunofluorescent staining; IHC, immunohistochemical staining; ICC, immunocytochemical staining
